# Can the Second to Fourth Digit Ratio (2D : 4D) Be a Marker to Determine Ankylosing Spondylitis Disease Activity?

**DOI:** 10.1155/2019/4612370

**Published:** 2019-02-11

**Authors:** Sevcan Uğur, Hasan Fatih Çay, İlhan Sezer, Cahit Kaçar

**Affiliations:** ^1^Department of Rheumatology, Balıkesir Atatürk City Hospital, Balıkesir, Turkey; ^2^Department of Rheumatology, Sağlık Bilimleri University, Antalya Education and Research Hospital, Antalya, Turkey; ^3^Department of Rheumatology, Faculty of Medicine, Akdeniz University, Antalya, Turkey

## Abstract

**Objective:**

The length ratio of the index finger (2D) to the ring finger (4D) (2D : 4D ratio) is considered a biomarker of prenatal sex hormone exposure. The 2D : 4D ratio is influenced by prenatal androgen and estrogen levels. Because ankylosing spondylitis (AS) influences men more frequently and severely than women, androgens are proposed to be related to AS pathogenesis. Estrogens have immune-modulating effects and reduce AS disease activity. The aim of this study was to assess the relationship between 2D : 4D ratio and AS disease activity.

**Material and Methods:**

In this study, 167 (43 female) patients diagnosed with AS were studied. The lengths of the second and fourth fingers were measured using a digital caliper. The 2D : 4D ratio was found by dividing the length of the second finger by the length of the fourth finger. AS disease activity was assessed with the Turkish version of the Bath Ankylosing Spondylitis Disease Activity Index (BASDAI). AS functional status was assessed with Bath Ankylosing Spondylitis Functional Index (BASFI). L-Schober, tragus to wall distance, finger to floor distance, and chest expansion were used to evaluate mobility.

**Results:**

In female patients, the right hand 2D : 4D ratios were higher than those in male patients. Biologic drug use was more frequent in males. The BASDAI scores were higher in female patients than in male patients. There were significant negative correlations between right and left hand 2D : 4D ratio and BASFI and BASDAI in female patients. There was no significant correlation between the 2D : 4D ratio and BASFI or BASDAI in male patients. We found a positive correlation between L-Schober and right hand 2D : 4D and a negative correlation between the left hand 2D : 4D ratio and finger to floor distance in female patients with AS.

**Conclusion:**

The 2D : 4D ratio of the right and left hand was low in female patients with high BASFI and BASDAI and low spinal mobility (L-Schober) was also linked to low female 2D : 4D. The lack of strong associations between 2D : 4D and AS in male patients may have resulted from their higher use of biologics.

## 1. Introduction

Ankylosing spondylitis (AS) is a chronic inflammatory disease and affects the axial skeleton leading to structural damage and functional limitation [1]. The ratio of the index finger (2D) length to the ring finger (4D) length (2D : 4D ratio) is considered a biomarker of prenatal sex hormone exposure [2]. The ratio of second and fourth digit lengths are highly influenced by the ratio of prenatal androgen to estrogen levels [3]. Because AS influences men more frequently and severely than women androgens are proposed to be related to AS pathogenesis. Although some studies have indicated that serum androgen levels are increased in AS patients compared to control groups, the role of sex steroids in AS pathogenesis is still poorly understood [4]. The 2D : 4D ratio is used to assess indirectly prenatal exposure to androgen [5]. In this study we aimed to investigate the relationship between right hand 2D : 4D ratio and AS disease activity and to determine whether 2D : 4D length ratio is a predictive marker for AS.

## 2. Materials and Methods

Patients diagnosed with AS according to the modified New York criteria were consecutively recruited in this study. The study was in accordance with the Declaration of Helsinki and was approved by the local Ethical Committee. The patients were signed and informed written consent to participate in the study. Patients with hand and finger deformity, finger amputation, hand arthritis, and with juvenile onset of AS were excluded from the study. Patients using right hand dominantly were included in the study. Demographic data such as age, sex, duration of disease, age at onset, family history, peripheral joint involvement, biological drug used, and uveitis were recorded. The functional status was assessed using Bath Ankylosing Spondylitis Functional Index (BASFI); BASFI score 4 and above was considered as a poor functional condition. The Turkish version of the Bath Ankylosing Spondylitis Disease Activity Index (BASDAI) was used to assess AS disease activity. Patients were grouped as having active (BASDAI score of 4 or more) and inactive (BASDAI score of less than 4) disease. L-Schober, chest expansion, fingertip to floor distance, and tragus to wall distances were measured to evaluate mobility. Tragus to wall distance was calculated by measuring the horizontal distance between the tragus and wall while standing with the heel and buttocks against to wall. Fingertip to floor distance was calculated by measuring the distance between the tip of right middle finger and the floor following maximal lumbar flexion. Chest expansion was appraised by measuring the difference of the circumferential distance of the thorax from the 4th intercostal level in maximum inspiration and maximum expiration. L-Schober was calculated by measuring the distance between the spina iliaca posterior superior and 10 cm apart in a standing position following maximal forward flexion.

The lengths of the second and fourth fingers were measured using a digital caliper with a resolution of 0.01 mm. Finger lengths were determined by measuring the distance between the proximal fold of the volar hand fingertip and the fingertip point. The right hand 2D : 4D ratio was found by dividing the measured length of the second finger by the length of the fourth finger.

### 2.1. Statistical Analysis

Data were analyzed using SPSS 16. *t*-test was used to analyze the differences between active and inactive disease. Pearson correlation test was used to evaluate the relationship between the 2D : 4D ratio and BASDAI, BASFI, L-Schober, chest expansion, tragus to wall distance, and fingertip to floor distance in female and male patients; *p* < 0.05 was considered significant. Regression diagrams of the BASDAI and right hand 2D : 4D ratio were made on Excel programme.

## 3. Results

In this study, 167 (43 female, 124 male) patients were included. The mean age was 41.41 ± 10.4 years. The onset of disease was earlier in male patients (*p* = 0.01). Characteristics of the patients are shown in Tables 1 and 2. The right hand 2D : 4D ratio was 0.987 ± 0.033 in patients. The right hand 2D : 4D ratios were 0.990 ± 0.036 in female patients and 0.986 ± 0.032 in male patients. The left hand 2D : 4D ratio was 0.981 ± 0.02 in patients. The left hand 2D : 4D ratios were 0.987 ± 0.031 in female patients and 0.978 ± 0.027 in male patients. The right and left hand 2D : 4D ratios were higher in female patients compared to male patients but this difference was not statistically significant. The mean BASDAI score was 2.9 ± 2.1. The mean BASDAI scores were 3.79 ± 2.22 in female patients and 2.64 ± 2.01 in male patients. In our study, 32.3% of the patients (52.5% female, 25% male) had high disease activity. The BASDAI score was higher in female patients. The disease activity was higher in female patients compared to male patients and the difference was significant (*p* = 0.002).

**Figure 1:**
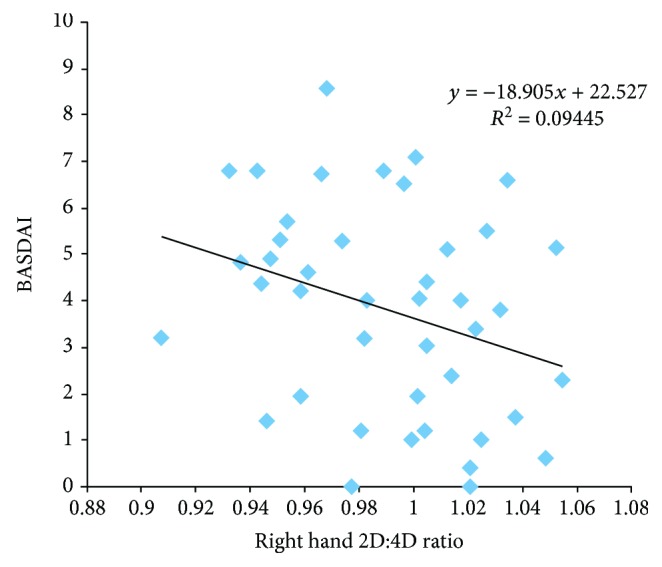


**Figure 2:**
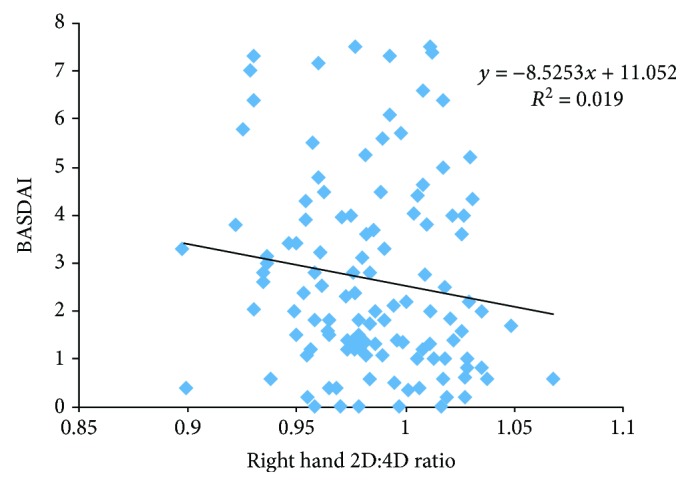


The right hand 2D : 4D ratio was higher (0.988 ± 0.033) in patients with low disease activity than in patients with high disease activity (0.985 ± 0.033) but this difference was not statistically significant. The right hand 2D : 4D ratio was 1.002 ± 0.035 in female patients with low disease activity and 0.980 ± 0.033 in female patients with high disease activity. Female patients with low disease activity had higher right hand 2D : 4D ratio (*p* = 0.04). The right hand 2D : 4D ratios were not statistically different in male patients with and without high disease activity. The right hand 2D : 4D ratios according to disease activity are shown in Table 3. There was no difference in the right hand 2D : 4D ratio in patients with and without uveitis and peripheral joint involvement. We found a negative correlation between the BASDAI and right hand 2D : 4D ratio (*p* = 0.03, *r* = −0.168). There was a negative correlation between the BASDAI and right hand 2D : 4D ratio in female patients (*p* = 0.04, *r* = −0.307). Although there was a negative correlation between the BASDAI and right hand 2D : 4D ratio in male patients, it was not statistically significant (*p* = 0.127, *r* = −0.138). The correlation of the right hand 2D : 4D ratio and BASDAI in female and male patients is shown in Figures 1 and 2. In our study, 66.5% of the patients were using biological drugs. The right hand 2D : 4D ratio was 0.985 ± 0.03 in patients using biologic drugs and 0.991 ± 0.03 in nonusers. The right hand 2D : 4D ratio was higher in patients not using biological drugs but this difference was not statistically significant. In our study, 79.6% of the patients had poor functional condition (BASFI ≥ 4). There was a negative correlation between the right hand 2D : 4D ratio and BASFI in female patients, and it was statistically significant (*p* = 0.01, *r* = −357). The mean L-Schober was 4.00 ± 1.71 in female patients and 3.28 ± 2.05 in male patients (*p* = 0.04). The fingertip to floor distance was lower in female patients compared to male patients (*p* = 0.004). The tragus to wall distance was longer in male patients than in female patients (*p* = 0.001). There was a negative correlation between the right hand 2D : 4D ratio and fingertip to floor distance and tragus to wall distance in female patients but it was not statistically significant. There was a positive correlation between the right hand 2D : 4D ratio and L-Schober in female patients (*p* = 0.005, *r* = 0.425). The right hand 2D : 4D ratio was positively correlated with L-Schober, fingertip to floor distance, and tragus to wall distance in male patients but it was not statistically significant.  Table 4 shows the L-Schober, fingertip to floor distance, tragus to wall distance, and chest expansion of patients. The correlations of the hand 2D : 4D ratio with different parameters in female and male patients are shown in Tables 5 and 6.

## 4. Discussion

The lengths of the second and fourth digits are sexually dimorphic [6]. While the second finger is usually shorter than the fourth finger in men, the second finger may be equal to or longer or shorter than the fourth finger in women. For this reason, while the 2D : 4D ratio is usually <1 in men, it may be either <1 or ≥ in women [7].

This difference in genders arises from intrauterine testosterone and estrogen balance [8]. While the index finger which is longer than the ring finger indicates the dominance of prenatal estrogen, the ring finger which is longer than the index finger indicates the dominance of prenatal testosterone hormone [9].

The 2D : 4D ratio is negatively correlated with testosterone and positively correlated with estrogen [10]. Lutchmaya et al. assessed the association between the 2D : 4D ratio and fetal testosterone (FT) and fetal estradiol (FE) which were measured from the amniotic fluid; they found a negative relationship between the right hand 2D : 4D ratio and FT/FE ratio [11]. Kyriakidis and Papaioannidou showed that the 2D : 4D ratio was significantly higher in females than in males [7, 12]. Previous studies reported that the ratio of the right hand 2D : 4D was more influenced by prenatal androgen compared to the left hand [9, 13]. For this reason, we used the right hand 2D : 4D ratio to evaluate the relationship between the 2D : 4D ratio and AS in our study. However, all patients in our study were using the right hand dominantly.

We found that the right hand 2D : 4D ratio was greater in female patients compared to male patients. The relationship between the 2D : 4D ratio and many diseases has been evaluated until today. Bilgic et al. compared male androgenic alopecia with healthy controls and found that the right hand 2D : 4D was lower in male with androgenic alopecia compared to healthy controls [8]. Wang et al. compared patients with coronary artery disease and healthy controls and found that the 2D : 4D ratio was lower in patients with coronary artery disease than healthy controls [14]. A meta-analysis reported that a low 2D : 4D ratio is associated with prostate cancer and brain tumors and a high 2D : 4D ratio is associated with breast cancer and cervical dysplasia. Moreover, it was noted that the high 2D : 4D ratio is associated with breast cancer and brain tumors being present at young age [2]. Doe et al. found that the 2D : 4D ratio was lower in patients with systemic lupus erythematosus than healthy controls and this result supported the exposure to high prenatal testosterone and low estrogen in SLE patients [15].

Although the relationship between the 2D : 4D ratio and a large number of diseases has been assessed to this day, as far as we know, there is no study evaluating the relationship between the 2D : 4D ratio and AS disease activity. To our knowledge, our study was the first study to evaluate the 2D : 4D ratio in patients with AS.

Previously, the male to female ratio was estimated to be 10 : 1 for AS patients but recent data showed that the ratio is 2–3 : 1 [16]. In our study, the male to female ratio was 2.88. This ratio was compatible with recent data. Male predominance, effect of pregnancy on development of AS, and immune modulating effects propose the role of sex steroids in AS [4]. The studies investigating the effects of androgens in AS showed different results. Although Masi stated that androgenicity increased in AS [17], Spector et al. did not show hyperandrogenicity in AS compared to healthy controls [18]. Tapia-Serrano et al. studied the testicular function by measuring hormone levels, seminal fluid analysis, and testicular reserve test in 22 patients with AS. In this study, nine male patients with severe active AS received biweekly 2,500 IU of human chorionic gonadotrophin (hCG) injections with a resulting increase in estradiol serum levels. When the values of estradiol reached 40 pg/ml or higher, a decrease in morning stiffness, lumbar pain, and sedimentation rate was observed in men treated with hCG. This study suggested a possible role of sex hormones in AS [19]. Sex steroid levels in female AS patients were investigated in a few studies. Giltay et al. investigated serum levels of sex steroids, luteinizing hormone, and sex hormone-binding globulin in patients with AS and in controls. In this study, only ten specimens of serum testosterone in women with AS were available [20]. Estrogen modulates immune-related processes [21]. The effects of estrogen on AS has been evaluated in a few studies. Jimenez-Balderas et al. revealed a decrease in arthritis and clinical activity in 17 female AS patients after oral estrogen therapy and found that estradiol levels were lower in menstruating patients with active AS compared to inactive AS. They also found that estrogen levels were lower in postmenopausal AS patients compared to controls [22]. A study performed on SKG mice showed that estrogen suppressed the development of arthritis in spondyloarthropathy. This study suggested that estrogen had an anti-inflammatory effect on the spondyloarthritis manifestations [23]. There have been many studies evaluating the relationship between the 2D : 4D ratio and adult androgen levels. Muller et al. found a negative correlation between the right-hand 2D : 4D ratio and testosterone levels in men [24]. In a study evaluating the relationship between the 2D : 4D ratio and testicular function, the ratio of 2D : 4D was found to be related to FSH [25]. There was a difference in the right hand 2D : 4D ratio between male and female patients in our study. But it was not statistically significant. So, these results may support the effect of androgens in the pathogenesis of AS especially in women. Many studies have shown that the BASDAI score, which is used as a disease activity scale, is higher in women. Roussou and Sultana found that the BASDAI score was higher in female patients with spondyloarthropathies than in male patients [26]. Similarly, Webers et al. found the BASDAI score was significantly higher in women with AS [27]. Ibn Yacoub et al. also found that the BASDAI score was higher in women [28]. Male patients have lower disease activity measured by BASDAI but they have worse spinal mobility and a more serious radiologic progression, and earlier starting of the disease in men [16]. In our study, the BASDAI score was lower in male patients and disease onset was earlier in men. It was compatible with literature. We found that the BASDAI score in female patients was significantly higher than that in male patients. In female patients, there was a negative correlation between the BASDAI score and the 2D : 4D ratio (*p* = 0.04, *r* = −0.307). When the 2D : 4D ratio increased, the BASDAI score was falling. There was also a negative correlation between the 2D : 4D ratio and BASDAI score in male patients but it was not significant. In male patients, this result may be caused because the vast majority of male patients were using biological drugs and only 25% of male patients had active disease. Patients using biologic drugs had a lower right hand 2D : 4D ratio than patients who did not use biologic drugs but the difference was not statistically significant. Our findings support that patients with a low right hand 2D : 4D ratio have a greater need to use biologic drugs compared to patients with a high right hand 2D : 4D ratio. We believe that this difference was not statistically significant because there were patients with active disease but have not used biological drugs yet. Our study showed that the disease activity and the need for biological medication is higher when the right hand 2D : 4D ratio is lower, especially in women. In our study, there was a significant negative correlation between the right hand 2D : 4D ratio and BASFI in female patients (*p* = 0.01, *r* = −0.357). There was also a negative correlation between the right hand 2D : 4D ratio and BASFI in male patients but it was not significant. In the presence of this finding, it may be considered that the 2D : 4D ratio can be used as a predictive indicator for activity and functional status in female patients with AS.

Thoracic and lumbar mobility is restricted in AS [29]. Larger tragus to wall distance shows worse spinal/upper cervical posture. Larger differences in L-Schober indicates greater lumbar movement, and smaller distance between the finger and floor shows higher spinal movement [30]. In our study, L-Schober was lower in male patients compared to female patients; on the other hand, the tragus to wall distance and finger to floor distance were higher in male compared to female patients. Our study showed that men had worse spinal mobility compared to women. We found a positive correlation between the right hand 2D : 4D ratio and L-Schober (*p* = 0.005, *r* = 0.425) in female patients. Our study showed that the higher 2D : 4D ratio is associated with higher lumbar mobility in female patients. Although it was not significant, there was a negative correlation between the right hand 2D : 4D ratio and tragus to wall distance and finger to floor distance in female patients. These results show that when the right hand 2D : 4D ratio increases, the ability of movement increases too. Our results support that the higher 2D : 4D ratio is a predictor of good spinal mobility in female patients.

According to our knowledge, our study was the first study that evaluates the relationship between the 2D : 4D ratios and AS. Our study showed that the 2D : 4D ratio may be used as a predictive activity indicator in female patients with AS. Further studies with bigger sample size and homogeneous patient groups in terms of treatment are necessary to confirm these findings.

## Figures and Tables

**Table 1:** Characteristics of the patients with ankylosing spondylitis. ^∗^*p* < 0.05.

	Total (*n* = 167)	Women (*n* = 43)	Men (*n* = 124)	*p*
Age, years	41.41 ± 10.40	42.72 ± 11.64	40.96 ± 9.95	*p* = 0.3
Disease durations, years	12.14 ± 8.26	10.19 ± 7.05	12.8 ± 8.56	*p* = 0.07
Age of disease onset	29.25 ± 9.73	32.52 ± 10.71	28.11 ± 9.15^∗^	*p* = 0.01
BASDAI	2.93 ± 2.12	3.79 ± 2.22	2.64 ± 2.01^∗^	*p* = 0.002
BASFI	2.48 ± 2.09	2.96 ± 2.27	2.31 ± 2.01	*p* = 0.08
Right 2D : 4D	0.987 ± 0.033	0.990 ± 0.036	0.986 ± 0.032	*p* = 0.4
Left 2D : 4D	0.981 ± 0.02	0.987 ± 0.031	0.978 ± 0.027	*p* = 0.08

**Table 2:** Special characteristics of the patients with ankylosing spondylitis.

	Total number/percentage	Women number/percentage	Men number/percentage
Family history	63/37.7%	21/48%	42/33.9%
Peripheral joint involvement	60/35.9%	17/39.5%	43/34.7%
Uveitis	26/15.6%	8/18.6%	18/14.5%
Use of the biological drugs	111/66.5%	22/51.2%	89/71.8%

**Table 3:** The 2D : 4D ratios in patients with active and inactive disease. ^∗^*p* < 0.05.

Right 2D : 4D	Active disease	Inactive disease	*p*
Women (*n* = 43)	1.002 ± 0.359 (*n* = 20)	0.980 ± 0.033^∗^ (*n* = 23)	*p* = 0.04
Men (*n* = 124)	0.985 ± 0.032 (*n* = 93)	0.988 ± 0.032 (*n* = 31)	*p* = 0.6
Total (*n* = 167)	0.988 ± 0.033 (*n* = 113)	0.985 ± 0.033 (*n* = 54)	*p* = 0.5

**Table 4:** The L-Schober, finger to floor distance, tragus to wall distance, and chest expansion of patients.

	Total (*n* = 167)	Women (*n* = 43)	Men (*n* = 124)	
L–Schober (cm)	3.47 ± 1.99	4.00 ± 1.71	3.28 ± 2.05^∗^	*p* = 0.04
Fingertip to floor distance (cm)	18.95 ± 15.37	13.22 ± 12.66	20.94 ± 15.77^∗^	*p* = 0.004
Tragus to wall distance (cm)	14.6 ± 5.5	12.8 ± 3.59	15.3 ± 5.9^∗^	*p* = 0.001
Chest expansion (cm)	2.9 ± 1.7	2.78 ± 1.19	3.05 ± 1.85	*p* = 0.3

**Table 5:** The correlations of right and left hand 2D : 4D ratio with age onset, L-Schober, finger to floor distance, tragus to wall distance, chest expansion, BASDAI, and BASFI in female patients.

	Right hand 2D : 4D ratio (*p*, *r*)	Left hand 2D : 4D ratio (*p*, *r*)
Age onset	(*p* = 0.973, *r* = −0.005)	(*p* = 0.589, *r* = 0.08)
L-Schober	(*p* = 0.005, *r* = 0.425)	(*p* = 0.325, *r* = 0.154)
Finger to floor distance	(*p* = 0.07, *r* = −0.278)	(*p* = 0.005, *r* = 0.420)
Tragus to wall distance	(*p* = 0.91, *r* = −0.16)	(*p* = 0.844, *r* = 0.03)
Chest expansion	(*p* = 0.96, *r* = 0.00)	(*p* = 0.77, *r* = 0.006)
BASDAI	(*p* = 0.04, *r* = −0.307)	(*p* = 0.004, *r* = −0.433)
BASFI	(*p* = 0.01, *r* = −0.357)	(*p* = 0.00, *r* = −0.522)

**Table 6:** The correlations of right and left hand 2D : 4D ratio with age onset, L-Schober, finger to floor distance, tragus to wall distance, chest expansion, BASDAI, and BASFI in male patients.

	Right hand 2D : 4D ratio (*p*, *r*)	Left hand 2D : 4D ratio (*p*, *r*)
Age onset	(*p* = 0.17, *r* = 0.122)	(*p* = 0.92, *r* = 0.009)
L-Schober	(*p* = 0.56, *r* = 0.52)	(*p* = 0.46, *r* = 0.06)
Finger to floor distance	(*p* = 0.57, *r* = 0.05)	(*p* = 0.403, *r* = −0.76)
Tragus to wall distance	(*p* = 0.99, *r* = 0.001)	(*P* = 0.04, *r* = 0.181)
Chest expansion	(*p* = 0.59, *r* = −0.49)	(*p* = 0.59, *r* = −0.04)
BASDAI	(*p* = 0.127, *r* = −0.138)	(*p* = 0.505, *r* = −0.06)
BASFI	(*p* = 0.187, *r* = −0.119)	(*p* = 0.98, *r* = 0.002)

## Data Availability

The data used to support the findings of this study are available from the corresponding author upon request.
